# Beyond Energy Metabolism: Exploiting the Additional Roles of NAMPT for Cancer Therapy

**DOI:** 10.3389/fonc.2019.01514

**Published:** 2020-01-17

**Authors:** Christine M. Heske

**Affiliations:** Pediatric Oncology Branch, National Cancer Institute, National Institutes of Health, Bethesda, MD, United States

**Keywords:** NAD^+^, NAMPT, sirtuins, PARP, ROS, cancer

## Abstract

Tumor cells have increased requirements for NAD^+^. Thus, many cancers exhibit an increased reliance on NAD^+^ production pathways. This dependence may be exploited therapeutically through pharmacological targeting of NAMPT, the rate-limiting enzyme in the NAD^+^ salvage pathway. Despite promising preclinical data using NAMPT inhibitors in cancer models, early NAMPT inhibitors showed limited efficacy in several early phase clinical trials, necessitating the identification of strategies, such as drug combinations, to enhance their efficacy. While the effect of NAMPT inhibitors on impairment of energy metabolism in cancer cells has been well-described, more recent insights have uncovered a number of additional targetable cellular processes that are impacted by inhibition of NAMPT. These include sirtuin function, DNA repair machinery, redox homeostasis, molecular signaling, cellular stemness, and immune processes. This review highlights the recent findings describing the effects of NAMPT inhibitors on the non-metabolic functions of malignant cells, with a focus on how this information can be leveraged clinically. Combining NAMPT inhibitors with other therapies that target NAD^+^-dependent processes or selecting tumors with specific vulnerabilities that can be co-targeted with NAMPT inhibitors may represent opportunities to exploit the multiple functions of this enzyme for greater therapeutic benefit.

## Introduction

**Figure 1:**
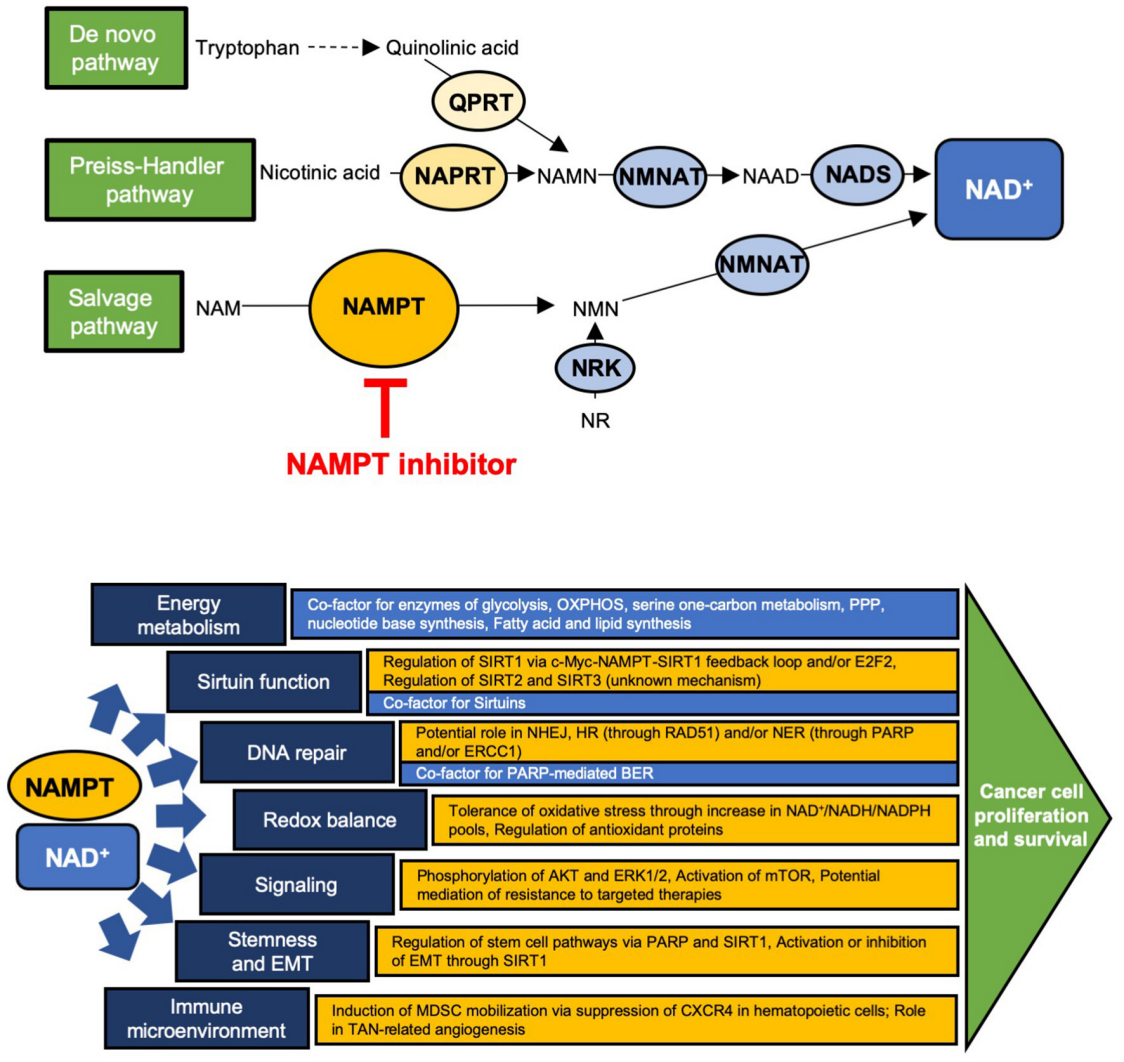
Schematic of the NAD^+^ production pathways and key enzymes and site of action of NAMPT inhibitors **(Top)** and the major downstream cellular functions of NAD^+^ (blue) and NAMPT (yellow) **(Bottom)**. **(Top)** QPRT, quinolinate phosphoribosyltransferase; NAPRT, nicotinate phosphoribosyltransferase; NAMPT, nicotinamide phosphoribosyltransferase; NMNAT, nicotinamide nucleotide adenylyltransferase; NRK, nicotinamide riboside kinase; NADS, NAD^+^ synthetase; NAMN, nicotinic acid mononucleotide; NAAD, nicotinic acid adenine dinucleotide; NAM, nicotinamide; NMN, nicotinamide mononucleotide; NR, nicotinamide riboside. **(Bottom)** OXPHOS, oxidative phosphorylation; PPP, pentose phosphate pathway; E2F2, E2F family member 2; NHEJ, non-homologous end joining; HR, homologous recombination; NER, nucleotide excision repair; BER, base excision repair; PARP, poly-ADP ribose polymerase; MDSC, myeloid-derived suppressor cell; TAN, tumor associated neutrophil.

Cancer cells have altered metabolic needs, including an accelerated rate of nicotinamide adenine dinucleotide (NAD^+^) cycling relative to normal cells (1). To maintain this, NAD^+^ metabolism is altered in cancer cells, many of which have an increased dependence on certain NAD^+^ production enzymes (2, 3) (Figure 1). Several redundant NAD^+^ production pathways exist. In the *de novo* pathway, tryptophan is first converted to quinolinic acid (QA) through a series of steps; QA is converted to nicotinic acid mononucleotide (NAMN) via quinolinate phosphoribosyltransferase (QPRT) and is then converted to NAD^+^ via nicotinamide nucleotide adenylyltransferase (NMNAT) and NAD synthetase (NADS). In normal cells, QPRT expression follows a tissue-specific distribution; more recent insights have revealed that QPRT expression is altered in some cancer cells (4–7). The Preiss-Handler pathway converts nicotinic acid (NA) to NAMN through nicotinate phosphoribosyltransferase (NAPRT), an enzyme that is widely expressed in normal tissues but variably expressed in cancer cells (8–11). NAMN is then converted to NAD^+^ through the activity of NMNAT and NADS, as in the *de novo* pathway. The salvage pathway, of which nicotinamide phosphoribosyltransferase (NAMPT) is the rate-limiting enzyme, converts nicotinamide (NAM) to nicotinamide mononucleotide (NMN), which is then converted to NAD^+^ through NMNAT. This pathway is of major importance to cancer cells, as it recycles NAM, the product of NAD^+^-consuming enzymes, back to NAD^+^. In fact, many types of cancer cells have been shown to highly express NAMPT, reflecting potentially increased dependence on this pathway due to high NAD^+^ utilization and in some cases, loss of expression of other key NAD^+^ biosynthetic enzymes (3, 9, 12, 13). Among the types of cancers reported to have high NAMPT expression are colorectal cancer (CRC), breast cancer, osteosarcoma, chondrosarcoma, pancreatic ductal adenocarcinoma, oral squamous cell carcinoma, prostate cancer, rhabdomyosarcoma, leiomyosarcoma, esophagogastric junction adenocarcinomas, thyroid cancer, leukemia, lymphoma, ovarian cancer, and some renal cancer, and in many of these, higher expression correlated with worse outcomes (14–30). Of note, NMN may also be produced from nicotinamide riboside via nicotinamide riboside kinase (9). Currently, however, NAMPT is the only NAD^+^ production enzyme that has been targeted in the clinic (2, 31, 32).

Clinical NAMPT inhibitors have investigated in a number of early phase clinical trials (Table 1). Published results on the early phase experience with first generation clinical NAMPT inhibitors describe a disease control rate of ~25% and few objective responses (33–38). Given the limited efficacy seen in these small studies, efforts to optimize the use of NAMPT inhibitors in the clinic are necessary. These include strategies such as drug combinations or selection of specific patient subsets more likely to be sensitive to these agents. Several new NAMPT inhibitors have recently entered early phase testing and preclinical efforts are focusing on use of these potential strategies to enhance activity and minimize toxicities (2, 3, 31, 32, 39).

**Table 1:** Summary of clinical trials testing NAMPT inhibitors.

**Agent**	**Phase**	**Cancer type**	**Administration**	**Patient number and age**	**Status**	**Primary objective**	**Reported DLTs**	**Reported responses**	**References**
CHS-828	1	Solid tumors	Oral Days 1–5 28 day cycle	16 (32–74)	Completed	RP2D: 20 mg/d × 5d q28d	Thrombocytopenia Thrombosis Esophagitis Diarrhea Constipation	No ORs 7 SD after 2 cycles	(33)
CHS-828	1	Solid tumors	Oral Day 1 21 day cycle	38 (30–70)	Completed	RP2D: 420 mg q21d	Thrombocytopenia Leukopenia Hematuria Diarrhea Mucositis	No ORs 11 SD	(34)
FK-866	1	Solid tumors	IV Continuous 96 h infusion (Days 1–4) 28 day cycle	24 (34–78)	Completed	RP2D: 0.126 mg/m^2^/hr	Thrombocytopenia	No OR 4 SD for at least 3 cycles	(35)
GMX-1777 (CHS-828 prodrug)	1	Advanced malignancies	IV continuous 24 hr infusion (Day 1) 21 day cycle	19 (median 57)	Completed	RP2D: 140 mg/m^2^/hr	Thrombocytopenia Hemorrhage Rash	No OR 5 SD	(36)
CHS-828	1	Solid tumors	Oral Days 1, 8, 15 28 day cycle	8 (51–73)	Premature closure	RP2D: Not defined	Diarrhea Fatigue Hypokalemia Hyperuricemia Dehydration Subileus Gastric ulcer	No OR	(37)
APO-866	2	Cutaneous T-cell lymphoma	IV 0.126 mg/m^2^/hr continuous 96 hr infusion (Days 1–4) 28 day cycle	14 (19–83)	Premature closure	Premature closure (lack of sufficient efficacy)	n/a	1 PR 6 SD	(38)
KPT-9274	1	Solid tumors or NHL	Oral Days 1, 3, 5, 8, 10, 12, 15, 17, 19, 22, 24, 26 28 day cycle (±niacin co-administration)	In process; 14 interim patients reported	In process NCT02702492	In process	Anemia	4 SD	(39)
OT-82	1	Lymphoma	Oral Days 1–3, 8–10, 15–17 28 day cycle	In process	In process NCT03921879	In process	In process	In process	n/a

*NHL, Non-Hodgkins lymphoma; RP2D, recommended phase 2 dose; OR, objective response; SD, stable disease*.

The role of NAD^+^ in supporting the energy metabolism of cancer cells has been well-established as NAD^+^ is an important co-factor for a number of metabolic enzymes (10). Accordingly, NAMPT inhibitors have been shown to impair energy metabolism through disruption of specific metabolic pathways, decreased ATP production, and increased energetic stress (40–44). In cancer cells, NAMPT inhibitors affect glycolysis (40, 45–55), oxidative phosphorylation (45, 49, 53, 56–59), serine biosynthesis and one-carbon metabolism (60), the pentose phosphate pathway (40, 53, 61), amino acid metabolism (53), purine and pyrimidine metabolism (53), and fatty acid and lipid synthesis (45, 62). In addition, cancer types harboring mutations in metabolic pathways, such as isocitrate dehydrogenase (IDH), have been shown to be exquisitely sensitive to loss of NAMPT activity (63–65).

Beyond its role in energy metabolism, NAD^+^ plays a vital role in many other cellular functions which may similarly be targeted with NAMPT inhibitors (13, 32, 66–70). An understanding of the non-metabolic implications of NAMPT inhibition may uncover additional targetable vulnerabilities, providing combinatorial opportunities for therapeutic intervention in cancer. The purpose of this review is to describe the impact of NAMPT and NAMPT inhibition on the non-energetic cellular functions of NAD^+^ in cancer cells.

## Sirtuin Function

The sirtuins are a family of NAD^+^-dependent deacylases and ADP-ribosyltransferases that are responsible for a significant amount of cellular NAD^+^ consumption (71). The role of sirtuins in cancer is increasingly being described and consequently, there is a growing understanding of how targeting sirtuin function may be beneficial in certain cancer types (72). Among these insights are an appreciation of the role of NAMPT in sirtuin expression (73). In CRC models, several groups have reported that regulation of SIRT1 activity is mediated by NAMPT (74–77). In some cases, *NAMPT* was found to be a direct transcriptional target of c-Myc, resulting in a positive feedback loop between c-Myc, NAMPT, and SIRT1 that drove tumor cell proliferation and progression (74–76). These studies showed that the use of NAMPT inhibitors resulted in a loss of SIRT1 expression, de-repression of *TP53*, and decreased tumor cell growth in CRC models (74, 75, 77). A similar effect has been observed in prostate (19) and gastric cancer models (78).

Regulation of the sirtuins by NAMPT has additionally been reported in other cancer types. In melanoma cells, NAMPT regulation of the E2F family member 2 was shown to impact transcription and translation of *SIRT1*, and genetic or pharmacologic loss of NAMPT activity resulted in activation of TP53 and apoptotic cell death (79). A similar regulatory network has been described in breast cancer cells where increased SIRT1 activity and induction of deacetylation of TP53 were observed upon exposure to the extracellular form of NAMPT (80).

In cancers where a direct regulatory link between NAMPT and the sirtuins has not yet been elucidated, experimental induction of NAMPT has been shown to have a corresponding effect on SIRT1 activity (81), while genetic or pharmacologic inhibition of NAMPT has resulted in decreased SIRT1 (82–85), SIRT2 (86), and SIRT3 (87). Interestingly, the functional significance of NAMPT on sirtuins is likely cancer cell type specific, since changes in SIRT1 expression with NAMPT inhibition have not been observed in all NAMPT inhibitor-sensitive cells (88). These differences may be important for clinical translation of NAMPT inhibitors, as they may play a role in determining which cancer types are more susceptible to NAMPT inhibitors (89).

## DNA Damage Repair Response

Poly-ADP ribose polymerases (PARPs) represent another group of NAD^+^-dependent proteins that consume a large proportion of cellular NAD^+^ (90). Among their functions, PARPs play a key role in DNA damage detection and repair (91). Thus, an expected consequence of NAD^+^ depletion is impaired DNA damage repair. Decreased PARP activity with NAMPT inhibitor use has been reported in several cancer models, forming the basis for preclinical testing of NAMPT inhibitors plus PARP inhibitors in Ewing sarcoma and triple-negative breast cancer (92, 93). In both studies, synergy between NAMPT inhibitors and PARP inhibitors was observed. In the breast cancer study, the effect was noted to be greatest in BRCA-deficient models, suggesting that underlying defects in homologous recombination (HR) may further enhance the efficacy of NAMPT inhibition (93). This is supported by data indicating that NAMPT inhibition impairs non-homologous end joining and increases cellular dependence on HR (94). In ovarian cancer models, a regulatory relationship between BRCA1 and NAMPT has been described (95). Although it is not BRCA-deficient, Ewing sarcoma is also characterized by defective HR (96), further supporting the idea that cancers with defective DNA repair mechanisms may have increased susceptibility to NAMPT inhibition. Interestingly, results from a recent study in preclinical leukemia models revealed functional antagonism between NAMPT and PARP inhibitors, suggesting that cell type specific differences in how these pathways interact may be present (97).

Other cancer types with DNA repair deficiencies have been identified as selectively sensitive to NAMPT inhibitors. Non-small cell lung cancers (NSCLC) with excision repair cross-complementation group 1 (ERCC1) deficiency were exquisitely sensitive to NAMPT inhibitors, *in vitro* and *in vivo* (98). ERCC1 deficiency is also associated with mitochondrial defects, suggesting that additional factors may contribute to NAMPT inhibitor sensitivity in this cancer subtype. In ovarian cancer, expression of NAPRT, the key enzyme in the Preiss-Handler pathway, correlated with a BRCA-ness gene expression signature, and cells carrying these features were more sensitive to NAMPT inhibitors (99). Mechanistic studies of the downstream consequences of NAMPT inhibitors on DNA damage repair have described a variety of effects including decreased PARylation (92, 99) decreased RAD51, and impaired double-strand break repair by the HR pathway (100).

Given these insights, a number of studies have sought to determine the efficacy of NAMPT inhibitors when combined with DNA damaging agents. Enhanced antitumor activity has been reported across multiple malignancies when genetic or pharmacological inhibition of NAMPT has been combined with radiation (101), DNA alkylating agents (63, 99, 100, 102, 103), topoisomerase inhibitors (19, 46, 86), or other classes of chemotherapy known to augment the effects of impaired DNA repair (19, 20, 43, 46, 104–106). Surprisingly, in some cancers, an improvement in efficacy was restricted to combinations with only certain chemotherapeutic agents, as in preclinical studies in pancreatic cancer models which revealed that gemcitabine, but not 5-fluorouracil (5-FU) or oxaliplatin, enhanced the antiproliferative effect of NAMPT inhibitors (107). In other studies, the combinatorial effects of NAMPT inhibitors with drugs from across chemotherapeutic classes was similar (46). The mechanisms for these differences are not known. Lastly, a study characterizing the effects of resistance to NAMPT inhibitors in CRC cell lines reported changes in expression of genes involved in DNA repair and an increased sensitivity to DNA damaging agents, further suggesting an intimate connection between NAMPT dependency and DNA damage repair (108). Taken together, these insights have clinical implications as they suggest that tumors with certain defects in DNA repair mechanisms may be selectively sensitive to NAMPT inhibitors, and that rational combinations with chemotherapies may enhance the efficacy of this class of agents, particularly in selected tumor types.

## Redox Homeostasis

Maintenance of intracellular redox homeostasis is a critical cellular process requiring a balance between reactive oxygen species (ROS) generation and elimination, as excessive levels of ROS can result in cell death (109). NAD^+^ is an important regulator of cellular ROS levels which can accumulate upon depletion of NAD^+^ (110). This is particularly true in cancer cells which generally have increased ROS production and require very tight control of ROS balance (111). Hence, an additional consequence of NAMPT inhibition is disruption of ROS homeostasis.

NAMPT has been shown to contribute to the cellular capacity to tolerate oxidative stress in a number of studies. In CRC cell lines, NAMPT functioned to increase NADH pools, protecting cells against oxidative stress (112). In breast cancer models, NAMPT increased the pool of NAD^+^ that could be converted to NADPH through the pentose phosphate pathway, thus maintaining glutathione in the reduced state. This was of particular importance in cells undergoing glucose-deprivation, for which high levels of NAMPT decreased mitochondrial ROS levels (61). Additionally, in several studies, NAMPT inhibition enhanced cancer cell susceptibility to oxidative stress through a reduction in antioxidative capacity via downregulation of antioxidant proteins (102, 113).

Depletion or inhibition of NAMPT has been shown to increase ROS in models of NSCLC (40), leukemia (43, 97), prostate cancer (19), breast cancer (61), glioblastoma (102), CRC (112), and others (85). In all cases, this was associated with a loss of cancer cell viability. In addition, there was a differential effect noted between the induction of ROS in cancer cells compared to normal cells treated with NAMPT inhibitors, suggesting the existence of a therapeutic window for ROS induction with these agents (114). Interestingly, not all cancer cells exhibit an increase in ROS upon inhibition of NAMPT. In Ewing sarcoma and some NSCLC models, cells were able to maintain ROS balance in the presence of a NAMPT inhibitor, suggesting that there may be cell-type dependent differences in these effects (40, 92). This may be the result of active compensatory NAD^+^ production pathways, such as the Preiss-Handler pathway, in certain cancers (114). A comprehensive understanding of these differences will be important to clinical translation of this class of agents as they may have implications for patient selection.

Finally, several studies have reported on the efficacy of combining NAMPT inhibitors with ROS inducing agents. Use of NAMPT inhibitors plus β-lapachone, an agent that targets the NADPH quinone oxidoreductase-1 (NQO1) and generates ROS, resulted in excessive ROS production and had enhanced efficacy against growth of pancreatic adenocarcinoma cells, particularly those which overexpressed NQO1 (52, 115). A similar effect was seen with this combination in NSCLC models (83). In addition, adding a NAMPT inhibitor to ROS-containing plasma-activated medium resulted in increased ROS production, decreased intracellular reduced glutathione, and cell death of breast cancer cells (116).

## Oncogenic Signaling

Crosstalk between NAMPT and oncogenic signaling pathways has been reported in several cancer models. From a clinical perspective, co-targeting NAMPT with these pathways may be a beneficial strategy. In some cases, oncogenic factors regulate expression and activity of NAMPT, such as in Ewing sarcoma, where the oncogenic transcription factor EWS-FLI1 has been shown to regulate *NAMPT* expression (49) and in breast cancer, where FOXO1, a tumor suppressor, negatively regulates the expression of *NAMPT* while AKT positively regulates it (117). In other cases, *NAMPT* regulates the activity of oncogenic signaling pathways. For example, *NAMPT* overexpression in breast cancer cells and extracellular NAMPT (eNAMPT) released by melanoma cell have both been associated with AKT phosphorylation (118, 119). Exogenous eNAMPT was also found to induce phosphorylation of AKT and ERK1/2 and increase proliferation of breast cancer cells, and the use of AKT and ERK1/2 inhibitors could abrogate these effects (120). In multiple cancer models, a decrease in phospho-ERK1/2 was observed with NAMPT inhibition (44, 121, 122) and combining NAMPT inhibitors with ERK1/2 blockade enhanced cell death (121).

An interaction between NAMPT and mTOR has also been described in a number of malignancies. In hepatocellular carcinoma cells, NAMPT inhibition was associated with loss of activation of mTOR and its downstream targets. A corresponding increase in AMPKα activation was also noted (41). A similar effect was observed in leukemia cells (42), pancreatic ductal adenocarcinoma cells (46), and pancreatic neuroendocrine tumor cells (123). In both pancreatic cancer subtypes, the antiproliferative effect of NAMPT inhibition could be potentiated with concurrent mTOR inhibitor treatment (46, 123). In multiple myeloma models, NAMPT inhibition was also associated with loss of mTOR activation which was thought to contribute to autophagic death (121). In contrast, changes in AMPKα and mTOR were not observed in studies of non-cancerous cells treated with NAMPT inhibitors (41).

A number of studies have investigated changes in NAMPT expression that occur with development of drug resistance to targeted therapies. Both in clinical samples and experimental models, BRAF inhibitor resistant melanoma cells expressed higher levels of both intra- and extracellular NAMPT than their sensitive counterparts (57, 124). Remarkably, BRAF inhibitor resistance could be overcome with addition of NAMPT inhibitors (57). In addition, induced expression of *NAMPT* was able to render melanoma cells resistant to BRAF inhibitors while BRAF inhibition in sensitive cells resulted in transcriptional downregulation of *NAMPT* (125).

In addition to the oncogenic signaling molecules already described, correlative studies have also proposed a link between NAMPT expression and EGFR (44, 126), HER2, and estrogen receptor positivity (127). Furthermore, based on the data supporting crosstalk between oncogenic signaling and NAMPT, co-targeting NAMPT along with other signaling pathway molecules, as has been described with the BTK inhibitor ibrutinib in Waldenstrom macroglobulinemia cells, could be a promising therapeutic strategy (128).

## Epithelial-Mesenchymal Transformation and Stemness

NAMPT has been described as a mediator of cancer cell stemness (129). Studies in clinical CRC tumor samples revealed that high *NAMPT* expression was associated with the presence of a high proportion of cancer-initiating cells. Mechanistically, this was the result of the influence of *NAMPT* on transcriptional regulation of stem cell signaling pathways and was mediated by SIRT1 and PARP (126, 130). In glioblastoma tumors and patient-derived stem-like cells, high *NAMPT* expression was observed (131). *NAMPT* overexpression in experimental models of glioblastoma resulted in a cellular phenotype consistent with that of a cancer stem-like cell (126), while pharmacological and genetic inhibition of NAMPT decreased the ability of glioblastoma stem cells to self-renew and form *in vivo* tumors (131). In one study, the loss of cancer stem cell pluripotency upon inhibition of NAMPT was the result of an excess of autophagy, a well-described consequence of NAMPT inhibition (15, 58, 64, 121, 132–135), which disrupted the maintenance of cancer cell stemness (136). NAMPT inhibition has also been shown to reverse the ability of cancer cells to dedifferentiate (137).

NAMPT inhibition also affects epithelial-mesenchymal transition (EMT) in cancer cells. In hepatocellular carcinoma cells, pharmacological NAMPT inhibition resulted in changes in EMT marker proteins indicating a reversal of EMT, as well as a reduction in cellular capacity for invasion and metastasis formation, through a decrease in SIRT1 (84). Similarly, data showing that both *NAMPT* overexpression and exogenous eNAMPT induced EMT in breast cancer cell lines (127), and that eNAMPT promoted osteosarcoma cell migration and invasion (138). Furthermore, NAMPT inhibition diminished motility in glioma cells (54). In contrast, in lung cancer cell lines, NAMPT inhibition activated EMT and increased cellular invasiveness also through decreased SIRT1 (85), and in breast cancer models, NAMPT inhibition enhanced metastatic behavior (139), suggesting the impact of NAMPT inhibition on EMT may be cell-type specific. Interestingly, expression of NAPRT, which differs across cancer cell lines, correlates with EMT status (140), and may be related to the differential effects of NAMPT inhibition on EMT. Thus, an understanding of the effect of NAMPT inhibition on metastasis is important as it may differ for different malignancies, impacting optimal clinical translation of these agents.

## Immune Regulation of Tumor Microenvironment

In addition to the effect of NAMPT on primary tumor cells, recent insights have begun to elucidate the impact of NAMPT on the immune suppressive characteristics of the tumor microenvironment (26, 141). In murine cancer models, macrophage colony stimulating factor was shown to increase NAMPT expression in myeloid cells which, in turn, negatively regulated CXCR4 expression in hematopoietic cells in the bone marrow. Consequently, low CXCR4 resulted in mobilization of immature myeloid-derived suppressor cells (MDSCs), contributing to tumor immunosuppression. Importantly, pharmacologic inhibition of NAMPT resulted in a decrease in MDSC mobilization, reversing the immunosuppression and re-sensitizing tumor cells to immunotherapeutic agents in preclinical models (142).

NAMPT has also been shown to be upregulated in tumor associated neutrophils (TANs) in patients with melanoma and head and neck cancer, and in murine cancer models. Inhibition of NAMPT in *ex vivo* TANs followed by adoptive transfer of the TANs into tumor-bearing mice reduced tumor angiogenesis and proliferation through suppression of *SIRT1* and resultant transcriptional blockade of pro-angiogenic genes (143). While more studies are required to better understand the role NAMPT inhibition plays in the microenvironmental immune milieu, these and other preliminary reports suggest NAMPT inhibitors could be used to enhance immunotherapies in the clinic (144). In addition, correlative studies describing the effects of NAMPT inhibition on tumor microenvironmental factors are currently lacking in the clinical literature but would be informative to further clinical development of this class of agents and should be pursued in future studies.

## Discussion

Given the critical role that NAD^+^ plays in the growth and survival of malignant cells, NAMPT is an attractive therapeutic target in cancer. In addition to its function in cellular energy metabolism, NAMPT is involved in sirtuin function, support of DNA repair mechanisms, maintenance of redox balance, molecular signaling, determination of cellular states, and tumor-related immune suppression. Depending on the cancer cell type, NAMPT inhibitors may be able to impair many of these additional functions. Furthermore, combination therapies with agents that target these functions in a complementary manner have the potential to dramatically improve the efficacy of NAMPT inhibitors. There are an increasing number of reports describing additive or synergistic effects of NAMPT inhibitors being used in combination with other agents in the preclinical setting. With newer generation NAMPT inhibitors currently undergoing phase 1 evaluation, clinical translation of these rational combinations is a logical next step.

In addition to developing rational combination regimens using NAMPT inhibitors, careful patient selection represents an additional opportunity to maximize the efficacy of these agents. For example, *IDH* mutant cancers have been shown to have exquisite sensitivity to NAMPT inhibitors (63–65), as have tumors deficient in NAPRT (9, 145–151). Patient selection may also be guided by recognition of specific vulnerabilities in the non-metabolic pathways supported by NAMPT, such as HR-deficiency or EMT targeting for metastatic disease. In conclusion, it is critical to understand the impact of NAMPT and NAMPT inhibitors on both the energetic and the non-energetic cellular functions of NAD^+^ in cancer as these insights may be key to future development of this class of agents.

## Author Contributions

CH researched, wrote, and edited the manuscript.

### Conflict of Interest

The author declares that the research was conducted in the absence of any commercial or financial relationships that could be construed as a potential conflict of interest.
